# Measles neutralising antibody levels in patients receiving intravenous immunoglobulin treatment – a sub-analysis of a randomized, cross-over bioequivalence trial

**DOI:** 10.1371/journal.pone.0316926

**Published:** 2025-02-07

**Authors:** Vatsala Rajendram, Martyn Paddick, John More

**Affiliations:** Bio Products Laboratory Ltd., Elstree, United Kingdom; Brigham and Women's Hospital, UNITED STATES OF AMERICA

## Abstract

**Background:**

Intravenous immunoglobulin is a replacement therapy for patients living with primary immunodeficiencies. Each batch of intravenous immunoglobulin is required by the Food and Drug Administration to contain threshold levels of measles neutralising antibodies. Widespread use of the measles vaccine has decreased measles antibody potency in the United States plasma supply. There is limited data on measles antibody trough levels in treated primary immunodeficiency patients. The aim of this sub-analysis was to evaluate the measles antibody trough levels in treated primary immunodeficiency patients.

**Methods:**

GMX07 was an open-label, two-period, crossover bioequivalence study which randomized 33 adult patients with primary immunodeficiency disease in 16 centres across the United States, the United Kingdom and Hungary. Eligible adult patients received five infusions of Gammaplex^®^ 5% followed by five infusions of Gammaplex^®^ 10%, or vice versa, on either a 21- or 28-day dosing regimen. The trial included 15 paediatric patients who were not randomized, receiving only five infusions of 10% product. This sub-analysis measured trough levels of measles neutralising antibodies using a Vero cell-based measles virus neutralisation assay.

**Results:**

Median measles antibody trough levels were ~ 1300 mIU/mL with no significant difference between Gammaplex 5% and Gammaplex 10% treatment (p >  0.9) or the 21-day or 28-day dosing regimen (p >  0.3). There was also no difference between mean measles neutralising antibody levels following Gammaplex 10% in adult or paediatric patients.

**Conclusions:**

Levels of measles neutralising antibodies in the 5% and 10% formulations of this intravenous immunoglobulin product provided protective antibodies well above accepted thresholds and were similar in adult and paediatric patients across both 21-day and 28-day dosing regimens. Switching between Gammaplex products did not affect antibody levels.

**Trial registration:**

ClinicalTrials.gov NCT01963143

## Introduction

Intravenous immunoglobulin (IVIG) is indicated as a replacement therapy in persons with primary immunodeficiencies (PI). For patients with PI, IVIG provides antibodies against a wide range of antigens and protects against serious bacterial infections and long-term infection-related complications, like pulmonary disease. [1,2]. While patients living with PI are not the only population who rely on immunoglobulin (IG) therapy, they are the primary community who depend on it for antibody replacement [3,4]. As such, IG products must meet minimum potency requirements, which, in the United States (US), include a specification for measles virus antibodies, defined in the Code of Federal Regulations subpart on Immune Globulin Human Food and Drug Administration regulation 21 CFR 640.104 [5]. While the seroprevalence of measles within the US population is high, widespread use of measles vaccinations can manifest as a reduction in naturally acquired measles neutralising antibodies in plasma donors, resulting in a decreased antibody potency [6–8]. Each IG product must have a minimum level of protective function against critical infectious diseases such as measles, diphtheria, and polio. Within the healthy population, the protective antibody level against measles is ≥ 120 mIU/mL [9]. In patients with PI, the protective measles antibody level is likely to vary depending on the type of immune deficiency and is currently unknown. With the decline in measles neutralising antibody potency, a protective measles antibody level of ≥ 240 mIU/mL, which is double the protective level against measles in the healthy population, has been suggested for all IG products [10]. A better understanding of the trough levels of measles neutralising antibodies in patients with PI receiving IVIG treatment is much needed.

The primary objective of this sub-analysis of the GMX07 trial was to measure the trough levels of measles neutralising antibodies in adult and paediatric patients receiving Gammaplex 5% and Gammaplex 10% IVIG to provide a benchmark of neutralising antibody transfer to PI patients. Secondarily, the impact on the levels of protective antibodies when switching between these IVIG products was also evaluated.

## Materials and methods

### Study design and patient population

GMX07 was an investigator-initiated, prospective, open-label, two-period, crossover bioequivalence study that has been described in detail previously. In brief, the trial included IVIG-treated patients with PI aged 16 to 55 years (adult cohort), or 2 to 15 years and ≥ 10 kg (paediatric cohort) (Clinical Trials NCT01963143) [11]. A stratified randomization list, where each infusion sequence was coded, randomized and then stratified by infusion schedule, was prepared by the Contract Research Organization (INC Research, LLC). Adult patients were randomized (1:1) before the first planned infusion (visit 1) to one of the following treatment sequences and stratified by dosing schedule (every 21 or 28 days): either five infusions of 5% IVIG followed by five infusions of 10% IVIG, or five infusions of 10% IVIG followed by five infusions of 5% IVIG. The first treatment sequence for each patient was deemed period 1, while the second treatment sequence was period 2. Paediatric patients were not randomized to treatment and received five infusions of 10% IVIG only. The 5% and 10% IVIG product were dosed at 300 to 800 mg/kg per infusion every 21 or 28 days. Participants returned for an end-of-study visit 28 days following the final study infusion. Depending on the frequency of infusion, the total study duration (screening to end-of-study visit) for any given adult patient was 38 to 48 weeks, and 23 to 28 weeks for any given paediatric patient.

### Neutralising measles antibody assay and testing of patient samples

On completion of each treatment period, trough level samples were taken immediately prior to infusion with IVIG and were tested for levels of neutralising antibody to measles using a Vero cell-based measles virus neutralisation assay, a type of tissue culture neutralizing dose (TCND50) assay developed by Bio Products Laboratory (BPL). The measles virus neutralisation assay was developed and validated in-house for testing patient samples as detailed below. The plasma samples were frozen and shipped to central testing labs. The reserve samples were dispatched to BPL laboratories for measles antibody potency testing. Samples were available from 32 adult patients receiving both 5% and 10% IVIG and from 15 paediatric patients receiving 10% IVIG only.

### Virus stock

A virus stock of measles virus (Strain Edmonston-Zagreb) was prepared using a master vial from the Health Protection Agency Culture Collections (HPACC), National Collection of Pathogenic Viruses (NCPV) Cat No 0007234v #897. A working stock was prepared in-house, with a titre of ~ 5.1 log Tissue Culture Infectious Dose 50% (TCID_50_)/mL.

### Vero cell line

The Vero cell line seed stock was purchased from the Health Protection Agency Cell Culture Collection (HPACC 84113001, Lot 10F019, passage 20 [p20]). This was sub-cultured to create a master stock (p28) and working stock (p34) which were frozen down in liquid nitrogen. Confluent culture of Vero cells was used to seed 96-well plates, with a seeding density of 1x10^5^ cells/mL.

### Reference immune serum globulin

To establish a reference standard in mIU/mL for the in-house measurement of the clinical samples, the Centre for Biologics Evaluation and Research, Food and Drug Administration (CBER-FDA) Reference Immune Serum Globulin (RISG), Lot no. 177 (10% solution, candidate reference) was used. Additionally, an in-house 10% immunoglobulin G (IgG) solution was calibrated against the WHO 3rd International Standard for Anti-Measles from the National Institute for Biological Standards and Controls (NIBSC) code 97/648, with an assigned unitage of 3000 mIU/mL. The endpoint results for the references were expressed in mIU/mL.

### Determination of measles neutralising antibody levels

The required sample vials were thawed at 37 °C ±  0.2 °C for ~ 5 to 15 minutes, dependent on the sample volume. The samples were then aliquoted and heated at 56 °C ±  0.5 °C for 30 minutes to inactivate the complement prior to starting the assay.

Heat inactivated sera and reference materials (in-house reference – 10% immunoglobulin) and calibrated CBER Lot 177 were serially diluted in minimum essential media and incubated with a fixed dose of 50 TCID_50_/mL measles virus for 1 hour at 36 °C ±  1 °C. A virus back titration plate was set up alongside, such that the working virus stock was serially titrated and assayed under the same conditions. The sample/virus mixture was transferred to pre-formed Vero cell monolayers (seeded at 1x10^5^ cells/mL in minimum essential media +  5% fetal calf serum) in a 96-well plate. Samples and references were incubated for 6 days at 36 °C ±  1 °C. At the end of the incubation period, the plates were fixed with 4% formal saline and stained with 0.1% Naphthalene black solution. The plates were scored for the number of uninfected wells in sample and reference plates and for a cytopathic effect in virus back titration plates.

The Vero cell monolayer displayed a cytopathic effect for those dilutions with insufficient antibodies to neutralise the measles virus. The endpoint titre was calculated using the Spearman-Kärber method (50% tissue culture neutralising dose). The neutralising antibody titre of the test samples was converted to mIU/mL by comparing the 50% endpoint of each sample to that of the in-house reference (10% immunoglobulin) with an assigned titre.

### Sample size determination

Upon recommendation by the United States Food and Drug Administration (FDA), a sample size of approximately 30 adult patients aged 16 to 55 years and 12 to 18 paediatric patients were chosen [12]. At least 16 evaluable adults were to be on a 28-day dosing regimen and at least 12 adults on a 21-day dosing regimen, and 4 to 6 paediatric patients were to be in each of the following age ranges: 2 to 5 years, 6 to 11 years, and 12 to 15 years. The upper age limit of 55 years was selected to reduce patient variability and is not reflective of any safety concerns for the use of Gammaplex in patients > 55 years old.

### Blinding

During the data collection phase, investigators had limited access to participants’ personally identifiable information, which was necessary to ensure proper follow-up and address any potential adverse events. All personally identifiable information was securely stored and managed in compliance with applicable data protection laws and the approved study protocol. This information was restricted to authorized investigators only.

Upon completion of the data collection phase, all personally identifiable information was removed from the dataset and each participant was assigned a unique identification code. Using this de-identified dataset, the investigators analysed the data and prepared the manuscript, ensuring participant anonymity.

### Ethical considerations

The protocol for the GMX07 trial was approved by an institutional review board or independent ethics committee for each of 16 study centres in the US (13 centres), the UK (2 centres), and Hungary (1 centre). The trial was conducted in accordance with the International Conference on Harmonisation of technical requirements for registration of pharmaceuticals for human use Harmonised Tripartite Guidelines for Good Clinical Practice, and each study participant (or their parent/guardian, if applicable) provided written informed consent to participate in the study [13].

### Statistical analysis

Statistical analysis of the data was carried out using Minitab (v17.2.1). Between-group comparisons were conducted with a Mann-Whitney U test, while comparisons on patients across treatment arms were conducted with a paired t-test. An interval plot was used to examine the mean change in measles neutralising antibody levels when patients switched Gammaplex treatment sequences (eg, five infusions of 5% IVIG, then five infusions of 10% IVIG). Significance was set at p <  0.05.

## Results

### Baseline patient characteristics

**Fig 1:**
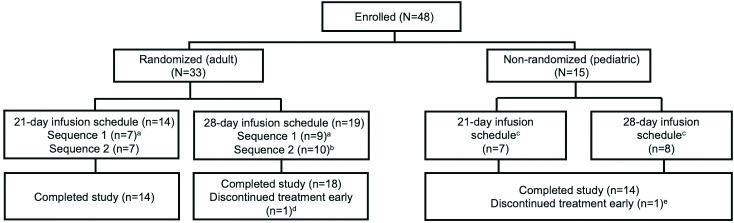
Patient disposition.

^a^Sequence 1: five infusions of Gammaplex 5% followed by five infusions of Gammaplex 10%. ^b^Sequence 2: five infusions of Gammaplex 10% followed by five infusions of Gammaplex 5%. ^c^Paediatric patients received five infusions of Gammaplex 10% only. ^d^One patient withdrew consent. One patient was withdrawn at the discretion of the investigator [11].

Between 12 March 2014 and 13 April 2016, 33 adult patients (39.5 ±  12 yrs; 12 males and 21 females) and 15 paediatric patients (9.6 ±  4.2 yrs; 8 males and 7 females) were enrolled into the study (Fig 1). Samples were available from 32 adult patients receiving Gammaplex 5% and 47 patients (32 adult and 15 paediatric) receiving Gammaplex 10%. One patient withdrew during the first infusion (Gammaplex 5%) and no results were recorded. Samples were taken following a minimum of five infusions (ie, at visit 6 and the follow-up visit) and were available from 24 of the 32 patients. The remaining patient samples taken from period 1 (one sample) or period 2 were earlier samples (ie, visit 4 or visit 9, rather than visits 5 and 10 respectively). All 15 paediatric patients who entered the study provided a sample for measles antibody analysis. Samples following a minimum of five infusions (ie, at the follow-up visit) were available from 10 of the 15 patients. An earlier sample was used for the five remaining paediatric patients, either at visit 4 or at visit 2. The trial ended when the final evaluable patient completed their end-of-study visit (28 days following the final study infusion).

Baseline characteristics of all patients are summarized in Table 1.

**Table 1:** Baseline characteristics of the study population.

Demographic characteristic	Adults (n = 33)	Paediatrics (n = 15)	All patients (N = 48)
**Age (years)**			
Mean (SD)	39.5 (11.99)	9.6 (4.15)	30.1 (17.29)
Median (range)	42.0 (17–55)	8.0 (3–15)	30.5 (3–55)
**Age group, n (%)**			
2-5 years	0	2 (13.3)	2 (4.2)
6-11 years	0	7 (46.7)	7 (14.6)
12-15 years	0	6 (40.0)	6 (12.5)
16-55 years	33 (100)	0	33 (68.8)
Female, n (%)	21 (63.6)	7 (46.7)	28 (58.3)
White, n (%)	33 (100)	15 (100)	48 (100)
**Diagnosis, n (%)**			
Common variable immunodeficiency	30 (90.9)	7 (46.7)	37 (77.1)
X-linked and autosomal forms of agammaglobulinemia	3 (9.1)	5 (33.3)	8 (16.7)
Hypogammaglobulinemia	0	0	0
**Baseline chest X-ray/CT scan, n (%)**			
Normal	28 (84.8)	15 (100)	43 (89.6)
Abnormal^a^	5 (15.2)	0	5 (10.4)
**Weight at screening (kg)**			
Mean (SD)	78.81 (20.3)	38.32 (16.0)	66.15 (26.8)
Median (range)	75.50 (51.6–140.0)	34.70 (14.8–65.4)	65.65 (14.8–140.0)

CT: computed tomography, CVID: common variable immunodeficiency, SD: standard deviation.

^a^Observed abnormal chest X-ray/CT scans included a possible emphysematous change or hyperplasia involving the right upper lobe (judged to be not clinically significant), a chronic left lower lobe bronchiectasis, an elevation of the left hemidiaphragm, a patient with radiographic findings suggestive of pulmonary fibrosis (patient had a history of CVID-related lung disease), and a patient with minimal parenchymal scarring with parenchymal configuration that suggested chronic air trapping (no evidence of acute disease was noted) [11].

### Establishment of an in-house reference for use with clinical samples

To establish a reference standard in mIU/mL, an in-house 10% IVIG batch was run alongside the NIBSC 3rd International Standard (IS) and the CBER-RISG 177 (candidate reference). This process was required as the NIBSC 3rd IS was found to be cytotoxic to cells at lower dilution (1/16 dilution). Measles antibody titres from 13 runs were used to calculate the value of the candidate reference standard in mIU/mL. Using the reference standard, the in-house 10% IgG units were 14,946 mIU/mL (range 12,801 to 17,666), CBER-RISG was 16,933 mIU/mL (range 12,227 to 19,822), and the NIBSC 3rd IS ranged from 794 to 5495 mIU/mL. Table 2 provides a summary of the conversion of TCND_50_ to mIU/mL. All product infused complied with the requirements set out in 21 CFR 640.104.

**Table 2:** Conversion of TCND_50_ to mIU/mL for the in-house reference.

	Endpoint Titre TCND_50_		
	10% IgG	CBER-RISG 177	3rd IS
**Average**	10,471	11,749	2089
**-3xSD**	3388	3236	794
^+^ **3xSD**	32,359	36,308	5495
**mIU/mL** ^a^	15,037	16,873	
**Range mIU/mL**	12,801–17,666	12,227–19,822	

^a^TCND_50_ of sample/TCND_50_ 3rd IS x 3000 mIU/mL. Note the assigned concentration for 3rd IS is 3000 mIU/mL.

### Effect of Gammaplex 5% and Gammaplex 10% on all patients

**Fig 2:**
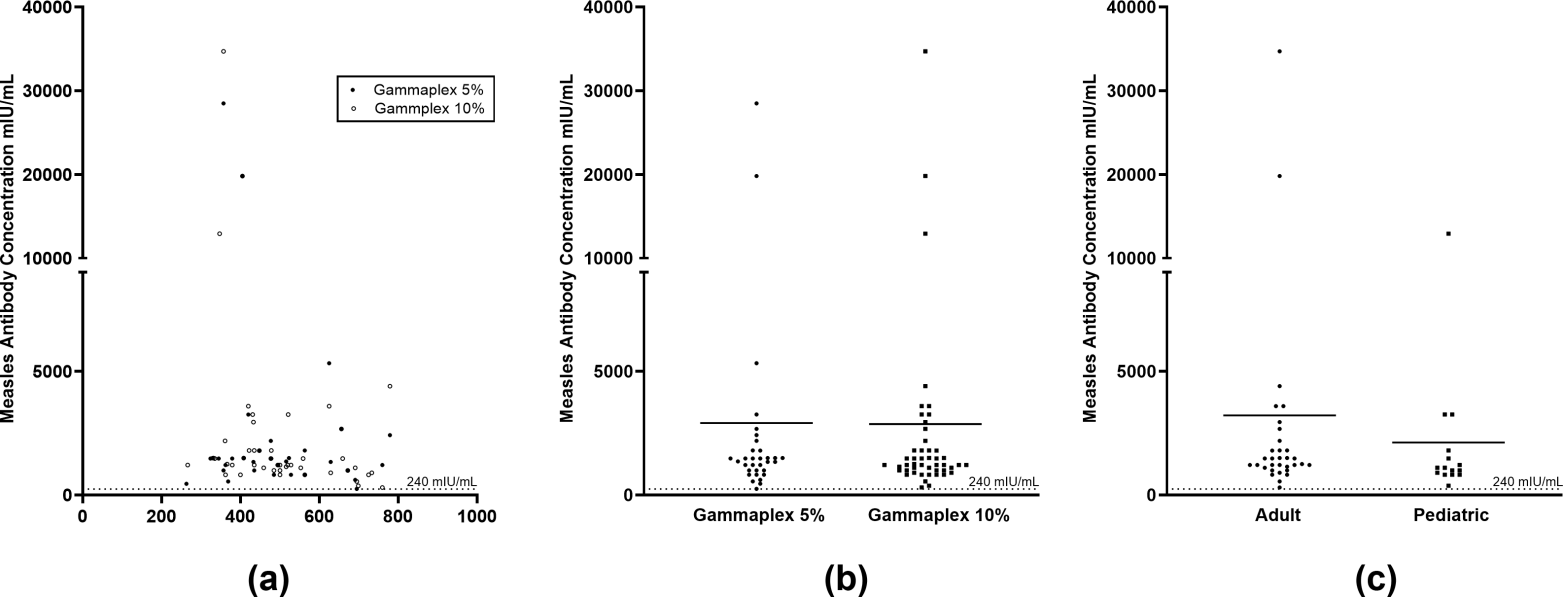
Trough measles neutralising antibody levels in PI patients receiving Gammaplex 5% or Gammaplex 10% IVIG treatment; (a) Trough measles antibody levels in all patients based on dose of 5% or 10% IVIG treatment; (b) Trough measles antibody levels in all patients by 5% or 10% IVIG treatment; (c) Trough measles antibody levels in adult and paediatric patients receiving 10% IVIG treatment.

Treatment with either five infusions of Gammaplex 5% or Gammaplex 10% IVIG resulted in an overall mean trough measles neutralising antibody concentration of 2882 mIU/mL (range 249.7 to 34,697 mIU/mL; Fig 2a; S1 Table). Following treatment with 5% IVIG, patients had a mean trough neutralising antibody concentration of 2908 mIU/mL (range 250 to 28,483 mIU/mL) and a 95% confidence interval of 833 to 4983 mIU/mL. After completion of 10% IVIG treatment, the mean trough neutralising antibody concentration for all patients was 2864 mIU/mL (range 304 to 34,697 mIU/mL) with a 95% confidence interval of 1178 to 4550 mIU/mL. Mean trough neutralising antibody concentrations were not significantly different following treatment with either 5% or 10% IVIG (p >  0.6; Fig 2b). Adults were treated with five infusions of both 5% and 10% IVIG in a randomized order. The mean trough neutralising measles antibody level following 10% IVIG treatment was 3213 mIU/mL (range 304 to 34,697 mIU/mL) with a 95% confidence interval of 816 to 5609 mIU/mL. There was no significant difference in measles antibody levels within all adult PI patients regardless of whether they were treated with 5% or 10% IVIG (p >  0.8; Fig 2b). Paediatric patients were only treated with 10% IVIG and had a mean trough measles neutralising antibody concentration of 2122 mIU/mL (range 371 to 12,936 mIU/mL) with a 95% confidence interval of 401 to 3842 mIU/mL. There was no significant difference in measles antibody levels between adult or paediatric PI patients receiving 10% IVIG (p >  0.1; Fig 2c).

### Switchover of treatment from 5% to 10% and from 10% to 5%

**Fig 3:**
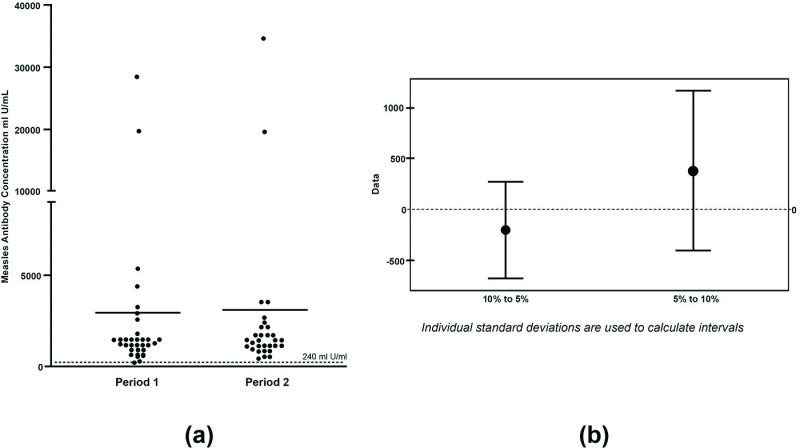
Measles neutralising antibody levels were not affected by order of Gammaplex IVIG treatment in adult PI patients; (a) Trough measles antibody levels in period 1 compared to period 2. Period 1 corresponds to the first round of five infusions of one product (i.e., 5% or 10% IVIG) and period 2 corresponds to the switch to the second round of five infusions of the second product (ie, 10% or 5% IVIG); (b) Confidence intervals for the mean change in measles neutralising antibody levels. Interval plot of the two treatment sequences: 10% to 5% IVIG and 5% to 10% IVIG. Confidence intervals are 95% of the mean. Paediatric patients only received 10% IVIG and are not included in either analysis.

Adult patients received both Gammaplex 5% and 10% treatment in a randomized treatment sequence. Period 1 corresponds to their first round of five infusions of one product and period 2 corresponds to the switch to the second round of five infusions of the second product. When examined within a patient, there was no significant difference in trough neutralising measles antibody levels between the two treatments, regardless of treatment order (p >  0.1; Fig 3a). The change in trough measles neutralising antibody levels for patients switching between products was further examined. The mean change in measles antibody levels was 127 mIU/mL (range -1973 to 6214 mIU/mL) with a 95% confidence interval of -342 to 597 mIU/mL. There was no evidence of any variations in trough measles neutralising antibody potency when switching between products (Fig 3b).

### Dosing schedule

**Fig 4:**
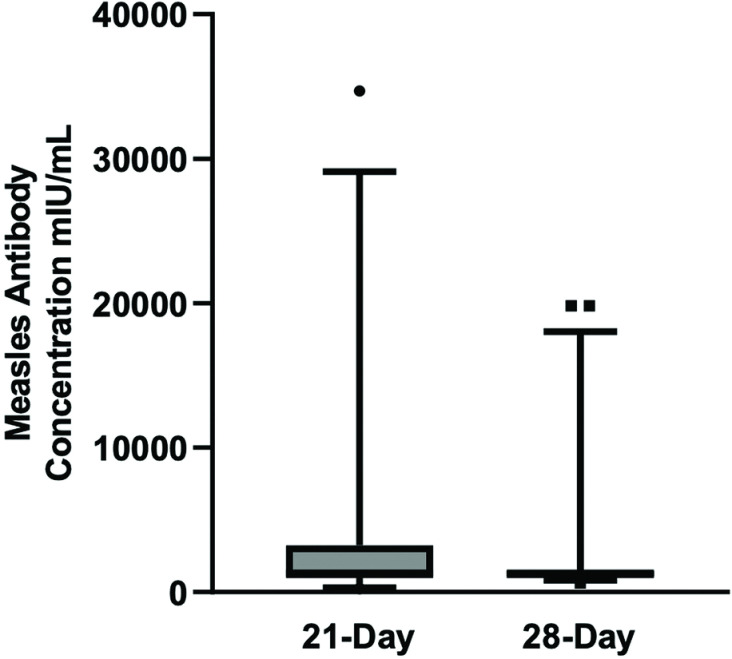
Comparison of 21-day to 28-day dosing schedule of both Gammaplex 5% and 10% in patients with PI. Whiskers of plot depict 5th and 95th percentiles.

Patients were treated with IVIG on either a 21-day or 28-day schedule, which was not randomized. Patients dosed on a 21-day schedule had a mean trough neutralising antibody level of 3688 mIU/mL (range 250 to 34,697 mIU/mL) with a 95% confidence interval of 1304 to 6072 mIU/mL. Patients who were treated on a 28-day schedule had comparable mean trough neutralising antibody levels of 2172 mIU/mL (range 607 to 19,806 mIU/mL) with a 95% confidence interval of 923 to 3422 mIU/mL. There was no significant difference in measles antibody levels whether patients received their treatment every 21 days or 28 days (p >  0.3; Fig 4).

## Discussion

The results presented here provide a benchmark of neutralising antibody transfer to patients with PI receiving IVIG treatment with Gammaplex 5% and Gammaplex 10%. Both Gammaplex products provided mean trough measles antibody titres substantially above the accepted thresholds for protection, and no titres fell below the protective thresholds. Importantly, mean measles neutralising antibody levels were not different in adult compared to paediatric patients with PI following 10% IVIG treatment. Patients receiving treatment at 21-day or 28-day intervals had equivalent trough levels of measles neutralising antibodies, and there was no effect of switching between Gammaplex products.

Patients living with PI depend on the protective effects of a variety of infectious disease antibodies within their IG products. However, the exact protective serum levels for measles virus prevention in patients with PI are not known. This is compounded by a lack of data describing measles neutralising antibody concentrations in this patient population, further limiting the understanding of the protective serum levels. For immunocompetent individuals, the protective measles neutralising antibody level is 120 mIU/mL [9] and the FDA has suggested that the protective level for patients living with PI should be higher (eg, 240 mIU/mL) [10]. Our results suggest that, on average, a patient with PI treated with either 5% or 10% IVIG would be expected to have a trough measles neutralising antibody level of approximately 1300 mIU/mL. Trough antibody levels represent the lowest level of seroprotective measles antibody levels, as they are measured at the point where circulating IG levels are at their lowest, just prior to infusion of the next dose [14]. The minimum trough value of IG measles neutralising antibody measured within this study (249.7 mIU/mL) was above the level established as being protective against the measles virus. Our results highlight that the increased protective level proposed for patients with PI is attainable following both 5% and 10% IVIG treatment. This data will provide additional support for understanding measles neutralising antibody protection in patients with PI.

Our results show no difference between measles neutralising antibody levels in adult or paediatric PI patients receiving 10% IVIG treatment. Measured trough levels are comparable to other studies examining measles neutralising antibody levels in both adult and paediatric patients with PI [14–16]. The PK of IG can be quite variable between patients, and a given dose of IVIG could result in different IG trough levels in different patients with similar body mass [17]. While there is limited literature examining the measles antibody levels in adult compared to paediatric patients, it is known that the PK and safety between adult and paediatric patients receiving IG treatment is similar with no difference in dosing requirements [11,14]. As such, it could be supposed that serum measles antibody concentrations would also be comparable and would be dose dependent. Patients treated with 10% IVIG, regardless of age, have similar protective levels of measles neutralising antibodies following IVIG treatment.

There were no statistically significant differences in trough measles antibody titres between patients with PI receiving IVIG treatment at 21-day or 28-day intervals (p >  0.3). It is common practice to provide IVIG treatment at 3- or 4-week intervals [12,18]. Following a 28-day (monthly) administration of IVIG, IG concentrations are back to baseline levels, which may decrease the protection of infection for some patients [18]. The mean trough antibody level for the 28-day treatment interval appears to be lower than what is measured following 21 days but is not statistically different. The range of antibody levels within the 21-day regimen has both the lowest and highest value, compared to the 28-day regimen. This highlights the heterogeneity within patients with PI, while also supporting the 28-day dosing regimen in providing adequate measles protection.

There were some limitations of this study. The sample size was small and may be most reflective of patients with common variable immunodeficiency (CVID), which could limit the generalizability of these results to all patients with PI. Of note, CVID is the most common of the primary immunodeficiencies and the sample approximates the distribution in the “real world”. Patients were excluded if they were over 55 years of age, pregnant or breastfeeding, or returned positive results for human immunodeficiency virus (HIV)-1, HIV-2, hepatitis C virus or hepatitis B surface antigen at screening. Particularly given that increasing age, pregnancy, and co-infection with HIV increase the risk of complications from measles infection [19–21], identifying and understanding any differences in levels of measles neutralising antibodies in these patient types, and subsequently, their risk of infection, may help guide a more targeted approach to care for these patient types. Future studies are warranted to accurately understand the differences in measles antibody trough levels in patients with PI receiving IVIG treatment.

Trough levels of measles neutralising antibodies in both Gammaplex 5% and Gammaplex 10% IVIG formulations provide protective antibodies well above accepted thresholds. This level of protection was similar in adult and paediatric patients across both 21-day and 28-day dosing regimens and was unaffected when switching between Gammaplex products.

## Supporting information

S1 Table Dataset of measles neutralising antibodies and the corresponding immunoglobulin trough levels. (DOCX)
