# Supplementary material for: Measles neutralising antibody levels in patients receiving intravenous immunoglobulin treatment – a sub-analysis of a randomized, cross-over bioequivalence trial
Source: PLoS One. 2025 Feb 7;20(2):e0316926. doi: 10.1371/journal.pone.0316926 (PMC11805365; doi:10.1371/journal.pone.0316926)
Supplement: S1 Table — (DOCX) [file pone.0316926.s001.docx]

S1 Table. Dataset of measles neutralizing antibodies and the corresponding immunoglobulin trough levels

| **Subject Group** | **Subject number** | **Dosing Regime** | **Infusion Visit** | **Treatment** | **Sample Date** | **Trough IgG**  **(g/L)** | **Measles neutralizing Ab (mIU/mL)** |
| --- | --- | --- | --- | --- | --- | --- | --- |
| Adult | 1001-01 | 28 | Infusion 6 | 10% IVIG | 04 SEP 2014 | 7.03 | 1211.13 |
| Adult | 1001-01 | 28 | Infusion 9 | 5% IVIG | 25 NOV 2014 | 7.85 | 1211.13 |
| Adult | 1001-02 | 28 | Infusion 6 | 5% IVIG | 24 SEP 2014 | 8.17 | 1492.41 |
| Adult | 1001-02 | 28 | Follow up | 10% IVIG | 11 FEB 2015 | 7.52 | 1211.13 |
| Adult | 1001-04 | 28 | Infusion 6 | 5% IVIG | 26 SEP 2014 | 7.13 | 1492.41 |
| Adult | 1001-04 | 28 | Infusion 9 | 10% IVIG | 19 DEC 2014 | 7.70 | 1492.41 |
| Adult | 1002-01 | 21 | Infusion 6 | 10% IVIG | 15 OCT 2014 | 9.61 | 1249.65 |
| Adult | 1002-01 | 21 | Follow up | 5% IVIG | 03 FEB 2015 | 8.94 | 550.04 |
| Adult | 1002-02 | 21 | Infusion 6 | 5% IVIG | 28 OCT 2014 | 9.47 | 1492.41 |
| Adult | 1002-02 | 21 | Follow up | 10% IVIG | 10 FEB 2015 | 9.49 | 1492.41 |
| Adult | 1003-01 | 28 | Infusion 6 | 10% IVIG | 20 OCT 2014 | 9.82 | 19805.74 |
| Adult | 1003-01 | 28 | Follow up | 5% IVIG | 09 MAR 2015 | 9.67 | 19805.74 |
| Adult | 1003-02 | 28 | Infusion 6 | 10% IVIG | 20 NOV 2014 | 13.16 | 1211.14 |
| Adult | 1003-02 | 28 | Follow up | 5% IVIG | 09 APR 2015 | 16.61 | 1475.34 |
| Adult | 1003-03 | 28 | Infusion 6 | 5% IVIG | 01 DEC 2014 | 10.34 | 1475.34 |
| Adult | 1003-03 | 28 | Follow up | 10% IVIG | 20 APR 2015 | 11.50 | 1797.17 |
| Adult | 1005-01 | 28 | Infusion 6 | 10% IVIG | 29 OCT 2014 | 9.51 | 1797.17 |
| Adult | 1005-01 | 28 | Infusion 10 | 5% IVIG | 18 MAR 2015 | 9.43 | 1797.17 |
| Adult | 1005-02 | 28 | Infusion 4 | 10% IVIG | 26 SEP 2014 | 7.90 | 1475.34 |
| Adult | 1005-02 | 28 | Follow up | 5% IVIG | 10 APR 2015 | 8.97 | 2189.23 |
| Adult | 1005-03 | 28 | Infusion 6 | 5% IVIG | 11 FEB 2015 | 8.58 | 1475.34 |
| Adult | 1005-03 | 28 | Follow up | 10% IVIG | 06 AUG 2015 | 8.32 | 2189.23 |
| Adult | 1005-04 | 28 | Infusion 6 | 5% IVIG | 13 MAR 2015 | 10.74 | 994.23 |
| Adult | 1005-04 | 28 | Follow up | 10% IVIG | 07 AUG 2015 | 8.30 | 1797.17 |
| Adult | 1007-01 | 28 | Infusion 6 | 10% IVIG | 16 JAN 2015 | 6.68 | 1475.34 |
| Adult | 1007-01 | 28 | Follow up | 5% IVIG | 05 JUN 2015 | 7.58 | 1475.34 |
| Adult | 1007-02 | 28 | Infusion 6 | 10% IVIG | 26 JAN 2015 | 8.21 | 1475.34 |
| Adult | 1007-02 | 28 | Follow up | 5% IVIG | 15 JUN 2015 | 8.23 | 1797.17 |
| Adult | 1008-01 | 21 | Infusion 4 | 5% IVIG | 16 MAY 2014 | 14.99 | 28483.37 |
| Adult | 1008-01 | 21 | Follow up | 10% IVIG | 20 OCT 2014 | 13.13 | 34696.96 |
| Adult | 1008-02 | 21 | Infusion 4 | 10% IVIG | 15 MAY 2014 | 14.70 | 4396.10 |
| Adult | 1008-02 | 21 | Follow up | 5% IVIG | 09 OCT 2014 | 14.01 | 2417.07 |
| Adult | 1008-03 | 21 | Infusion 6 | 10% IVIG | 17 JUN 2014 | 9.63 | 2944.37 |
| Adult | 1008-03 | 21 | Follow up | 5% IVIG | 29 SEP 2014 | 9.34 | 1336.87 |
| Adult | 1008-04 | 21 | Infusion 6 | 5% IVIG | 30 JUN 2014 | 14.05 | 5322.21 |
| Adult | 1008-04 | 21 | Follow up | 10% IVIG | 14 OCT 2014 | 13.82 | 3586.67 |
| Adult | 1008-05 | 21 | Infusion 6 | 5% IVIG | 03 JUL 2014 | 10.75 | 1354.29 |
| Adult | 1008-05 | 21 | Follow up | 10% IVIG | 22 DEC 2014 | 10.30 | 1139.16 |
| Adult | 1008-06 | 21 | Infusion 6 | 10% IVIG | 14 JUL 2014 | 14.03 | 2667.42 |
| Adult | 1008-06 | 21 | Follow up | 5% IVIG | 27 OCT 2014 | 14.15 | 2667.42 |
| Adult | 1008-07 | 21 | Infusion 6 | 5% IVIG | 28 AUG 2014 | 13.62 | 3249.32 |
| Adult | 1008-07 | 21 | Follow up | 10% IVIG | 12 DEC 2014 | 12.54 | 3586.67 |
| Paediatric | 1008-08 | 21 | Follow up | 10% IVIG | 24 NOV 2014 | 10.74 | 12935.75 |
| Paediatric | 1008-09 | 21 | Follow up | 10% IVIG | 11 DEC 2014 | 7.93 | 3249.3 |
| Adult | 1008-10 | 21 | Infusion 6 | 5% IVIG | 11 DEC 2014 | 8.29 | 249.74 |
| Adult | 1008-10 | 21 | Follow up | 10% IVIG | 25 MAR 2015 | 9.02 | 550.04 |
| Paediatric | 1008-11 | 21 | Follow up | 10% IVIG | 16 FEB 2015 | 9.13 | 1797.17 |
| Paediatric | 1008-12 | 21 | Follow up | 10% IVIG | 08 APR 2015 | 9.34 | 3249.30 |
| Paediatric | 1008-13 | 21 | Follow up | 10% IVIG | 14 SEP 2015 | 9.71 | 1097.47 |
| Paediatric | 1008-14 | 21 | Follow up | 10% IVIG | 28 AUG 2015 | 8.39 | 1097.47 |
| Paediatric | 1009-01 | 28 | Follow up | 10% IVIG | 21 SEP 2015 | 11.48 | 900.94 |
| Paediatric | 1009-02 | 21 | Infusion 4 | 10% IVIG | 09 JUL 2015 | 7.74 | 370.67 |
| Adult | 1010-01 | 28 | Infusion 6 | 5% IVIG | 11 SEP 2014 | 9.37 | 816.19 |
| Adult | 1010-01 | 28 | Follow up | 10% IVIG | 27 FEB 2015 | 11.62 | 816.19 |
| Adult | 1010-02 | 21 | Infusion 6 | 10% IVIG | 04 AUG 2014 | 13.44 | 304.30 |
| Adult | 1010-02 | 21 | Follow up | 5% IVIG | 08 DEC 2014 | 13.13 | 1211.41 |
| Adult | 1010-03 | 28 | Infusion 6 | 10% IVIG | 02 OCT 2014 | 9.23 | 994.24 |
| Adult | 1010-03 | 28 | Follow up | 5% IVIG | 19 FEB 2015 | 10.16 | 994.24 |
| Adult | 1010-05 | 28 | Infusion 6 | 5% IVIG | 14 OCT 2014 | 9.15 | 1336.87 |
| Adult | 1010-05 | 28 | Follow up | 10% IVIG | 03 MAR 2015 | 9.54 | 900.94 |
| Adult | 1010-06 | 28 | Infusion 6 | 5% IVIG | 04 NOV 2014 | 9.99 | 607.14 |
| Adult | 1010-06 | 28 | Infusion 9 | 10% IVIG | 26 JAN 2015 | 10.87 | 1097.47 |
| Adult | 1010-07 | 28 | Infusion 6 | 5% IVIG | 29 DEC 2014 | 11.23 | 816.19 |
| Adult | 1010-07 | 28 | Follow up | 10% IVIG | 18 MAY 2015 | 9.80 | 994.24 |
| Paediatric | 1010-08 | 28 | Follow up | 10% IVIG | 05 NOV 2014 | 8.46 | 1475.67 |
| Adult | 1010-09 | 28 | Infusion 6 | 5% IVIG | 05 FEB 2015 | 8.98 | 816.19 |
| Adult | 1010-09 | 28 | Follow up | 10% IVIG | 25 JUN 2015 | 8.29 | 1211.41 |
| Paediatric | 1012-02 | 28 | Infusion 4 | 10% IVIG | 13 FEB 2015 | 10.93 | 816.19 |
| Paediatric | 1013-02 | 21 | Follow up | 10% IVIG | 23 NOV 2015 | 9.23 | 816.19 |
| Paediatric | 1014-02 | 21 | Infusion 4 | 10% IVIG | 18 FEB 2015 | 12.59 | 994.24 |
| Paediatric | 1015-01 | 28 | Infusion 2 | 10% IVIG | 18 SEP 2015 | 10.23 | 994.24 |
| Paediatric | 1016-01 | 28 | Infusion 4 | 10% IVIG | 08 JUL 2015 | 7.26 | 816.19 |
| Adult | 2001-01 | 21 | Infusion 4 | 5% IVIG | 20 OCT 2014 | 8.88 | 1211.13 |
| Adult | 2001-01 | 21 | Follow up | 10% IVIG | 16 MAR 2015 | 9.32 | 816.20 |
| Adult | 2002-01 | 21 | Infusion 6 | 5% IVIG | 04 NOV 2014 | 8.27 | 994.24 |
| Adult | 2002-01 | 21 | Follow up | 10% IVIG | 10 MAR 2015 | 9.53 | 1211.13 |
| Adult | 2002-02 | 21 | Infusion 4 | 10% IVIG | 14 OCT 2014 | 8.18 | 1211.13 |
| Adult | 2002-02 | 21 | Follow up | 5% IVIG | 10 MAR 2015 | 6.93 | 451.54 |
| Paediatric | 3001-01 | 28 | Follow up | 10% IVIG | 23 JUN 2015 | 6.67 | 1211.13 |

Ab, antibody; IgG, immunoglobulin G; IVIG, Intravenous immunoglobulin.
